# Supplementary material for: Effect of 3-Hydroxyvalerate Content on Thermal, Mechanical, and Rheological Properties of Poly(3-hydroxybutyrate-co-3-hydroxyvalerate) Biopolymers Produced from Fermented Dairy Manure
Source: Polymers (Basel). 2022 Oct 3;14(19):4140. doi: 10.3390/polym14194140 (PMC9571417; doi:10.3390/polym14194140)
Supplement: Supplementary file 1 [file polymers-14-04140-s001.zip › polymers-1920304-supplementary.pdf]

Effect of 3-hydroxyvalerate content on Thermal, Mechanical, and Rheological properties of Poly(3-hydroxybutyrate-co-3-hydroxyvalerate) biopolymers produced from fermented dairy manure

Maryam Abbasi [1], Dikshya Pokhrel [1], Erik R. Coats [2], Nicholas M. Guho [2] and Armando G. McDonald [1,*]

[1] Department of Forest, Rangeland and Fire Sciences, University of Idaho, Moscow, ID, USA
[2] Department of Civil and Environmental Engineering, University of Idaho, Moscow, ID, USA
* Correspondence: armandm@uidaho.edu; Tel.: +1-2088859454

Supplemental Material

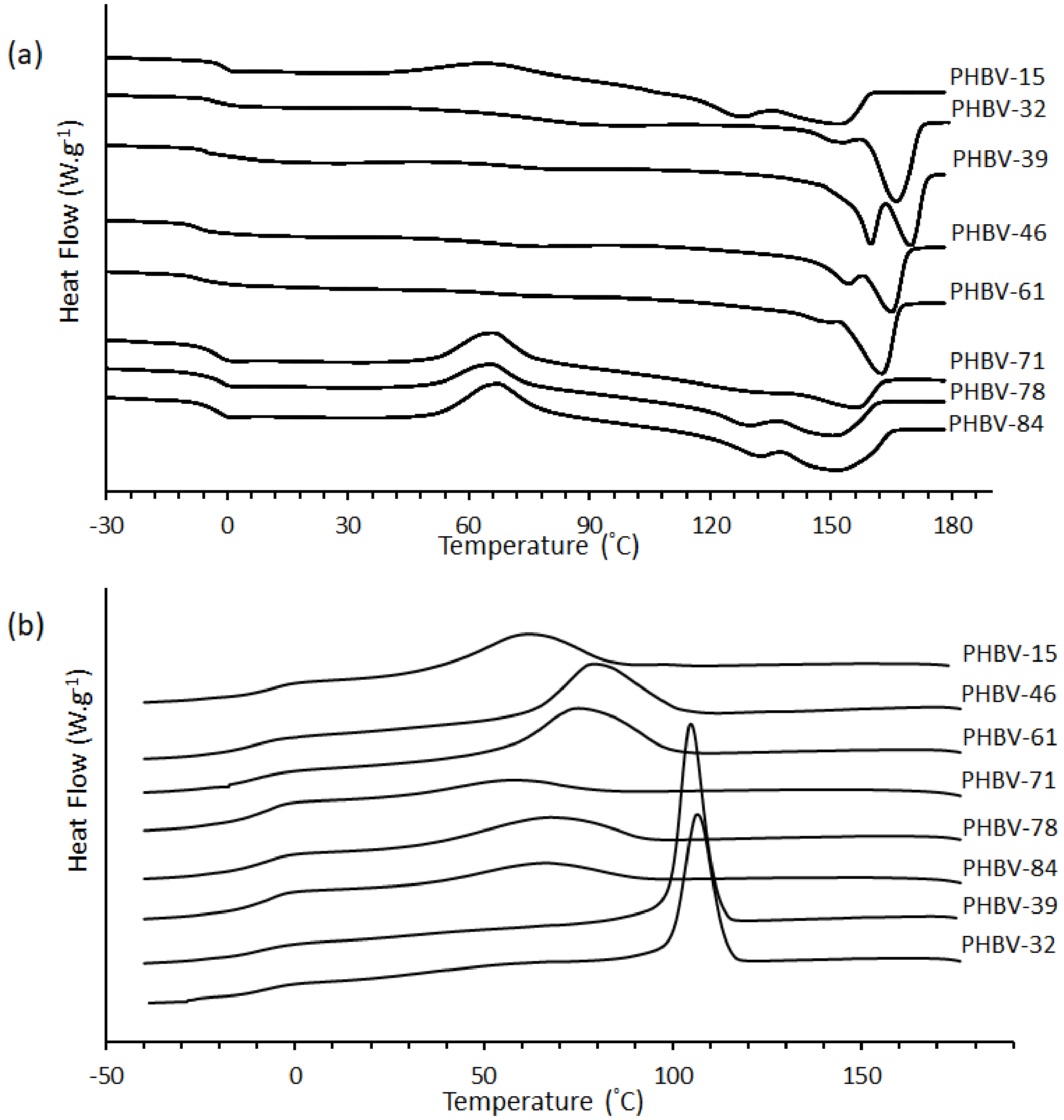

Figure S1. DSC thermograms for pure PHBV-15 to PHBV-84, (a) second heating scan; and (b) first cooling scan.

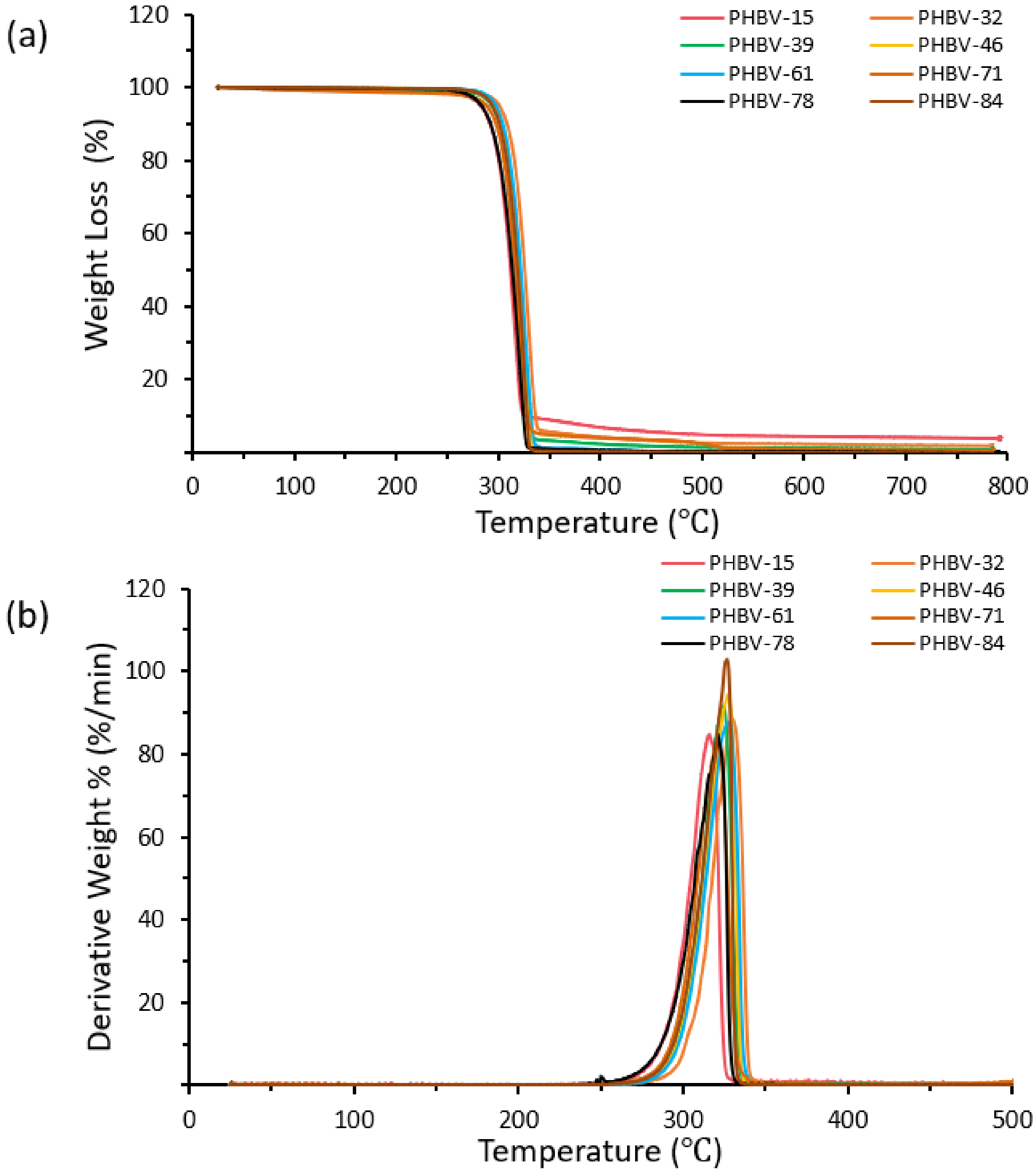

Figure S2. (a) TGA thermograms; and (b) DTG thermograms for pure PHBV-15 to PHBV-84.

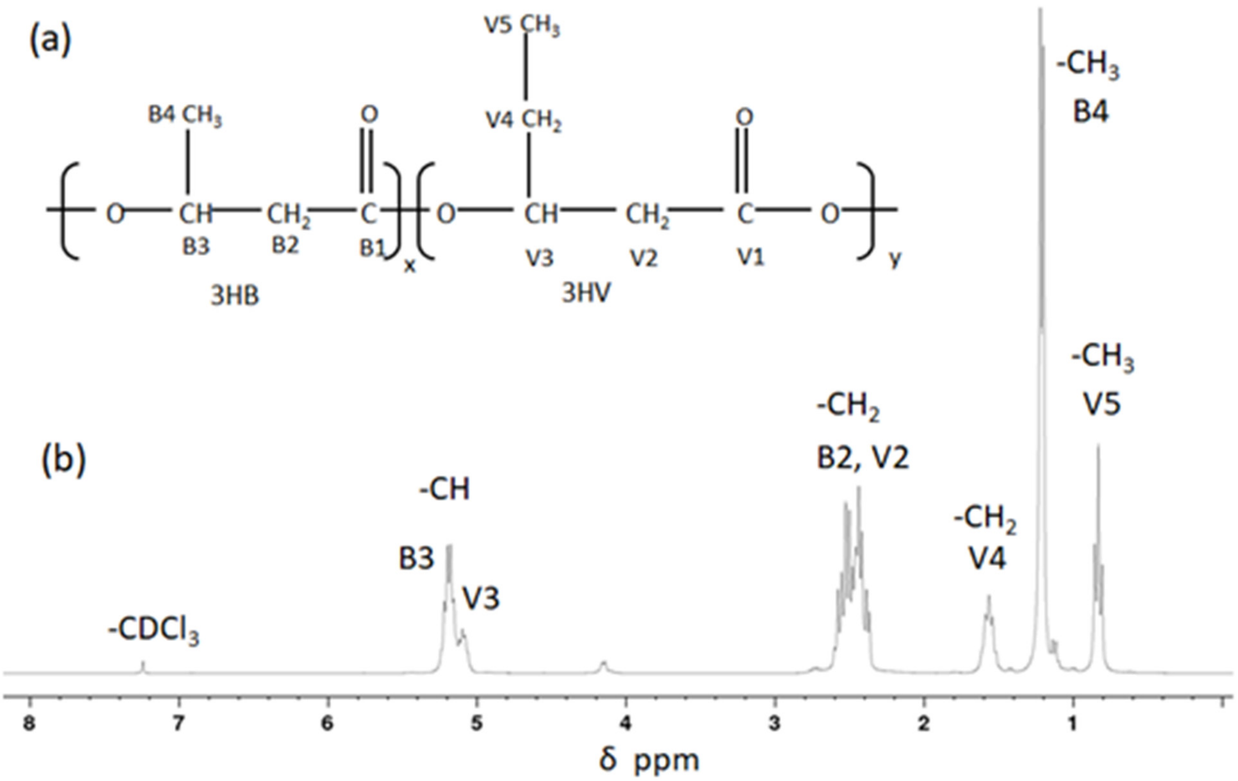

Figure S3. ^{1}H-NMR spectrum of PHBV-61, (a) structure; and (b) peak assignments.

Table S1. ^{13}C-NMR chemical shifts for carbonyl and methylene carbons in PHBV operational day 61.

Functional group	Chemical Shift (δ, ppm)	Sequence
C=O (B1, V1)	169.56	V*V
	169.38	B*V
	169.35	V*B
	169.18	B*B
CH_2 (V2)	38.86	BV*B
	38.83	VV*B
	38.73	BV*V
	38.71	VV*V
CH_2 (B2)	40.87	B*B
	40.83	B*V
CH_2 (V4)	26.91	BV*B
	26.88	VV*B
	26.85	BV*V
	26.82	VV*V

The coefficient of R is defined as [1]:

$R = \frac{L_V^R}{L_V^E} = \frac{L_B^R}{L_B^E}$ (Eq S1)

L_V^E and L_B^E indicate the number average lengths of HV and HB blocks in the copolymers on the experimental triad level which can be calculated by the following equations:

$$L_V^E = \frac{(F_{VVV}+F_{VVB}+F_{BVV}+F_{BVB})}{(F_{BVB}+F_{VVB})} \quad \text{(Eq S2)}$$

$$L_B^E = \frac{(F_{BBB}+F_{VBB}+F_{BBV}+F_{VBV})}{(F_{VBV}+F_{VBB})}$$

F_{XYZ} shows the relative molar fraction of XYZ triad sequence. L_V^R and L_B^R are the number average lengths of HV and HB blocks gained from HB and HV units which are statistically random distributed in the copolymer and are defined by following equations:

$$L_B^R = K + 1 \quad \text{(Eq S3)}$$

$$L_V^R = \frac{(K+1)}{K}$$

$$K = \frac{[HB]}{HV}$$

$$[HB] = \frac{K}{(K+1)}$$

$$[HV] = \frac{1}{(K+1)}$$

K is the ratio between the concentration of HB and HV units in the copolymer [1].

The diad and triad sequence distribution of PHBV biopolymer samples were calculated using three models as following [2,3]:

(i) Bernoullian statistics model, the simplest random copolymer model, which describes a statistically random copolymer. Calculation was expressed with the experimental mole fraction of 3HV unit (F_V^E).

$$F_{VV} = (F_V^E)^2 \quad \text{(Eq S4)}$$

$$F_{VB} = F_{BV} = F_V^E\,(1 - F_V^E)$$

$$F_{BB} = (1 - F_V^E)^2$$

$$F_{VVV} = (F_V^E)^3$$

$$F_{BVV} = F_{VVB} = (F_V^E)^2\,(1 - F_V^E)$$

$$F_{BVB} = F_V^E\,(1 - F_V^E)^2$$

Where F_{VV}, F_{VB}, F_{BV}, and F_{BB} (F_{XY} represents the mole fraction of XY sequence). The parameters associated with the superscript E shows the values that can be determined experimentally.

(ii) First-order Markovian model that is applicable on block, random and alternative copolymers. The relations can be described as follows:

$$P_{VV} = \frac{F_{VV}^E}{F_V^E} \quad \text{(Eq S5)}$$

$$P_{VB} = \frac{F^E_{VB}}{F^E_V}$$

$$P_{BV} = \frac{F^E_{BV}}{F^E_B}$$

$$P_{BB} = \frac{F^E_{BB}}{F^E_B}$$

$$F_{VV} = \frac{P_{VV}\ P_{BV}}{(P_{VB} + P_{BV})}$$

$$F_{VB} = F_{BV} = \frac{P_{VB}\ P_{BV}}{(P_{VB} + P_{BV})}$$

$$F_{BB} = \frac{P_{VB}\ P_{BB}}{(P_{VB} + P_{BV})}$$

$$F_{VVV} = \frac{P^2_{VV}\ P_{BV}}{(P_{VB} + P_{BV})}$$

$$F_{BVV} = F_{VVB} = \frac{P_{VV}\ P_{BV}\ P_{VB}}{(P_{VB} + P_{BV})}$$

$$F_{BVB} = \frac{P^2_{VB}\ P_{BV}}{(P_{VB} + P_{BV})}$$

Where P_{ij} *(i=j= B* or *V)* is the conditional possibility of *j* addition to the *i* last unit at the propagating chain end *i* with the relations that $P_{BV}+P_{BB}=1$ and $P_{VB}+P_{VV}=1$

(iii) A mixture of two Bernoullian random copolymers. If two Bernoullian model copolymers with the 3HV mole fractions of *A* and *B* are mixed with a molar ratio of *X:(1-X)*, then the three values of *A*, *B*, and *X* can be determined from the molar fractions of 3HV-centered triad sequences through the following equations using the Newton Method and MATLAB R2014a software. *A, B, X* in these equations must be between 0 and 1.

$$F^E_{BVV} = F^E_{VVB} = A^2(1-A)X + B^2(1-B)(1-X) \qquad \text{(Eq S6)}$$

$$F^E_{BVB} = A(1-A)^2X + B(1-B)^2(1-X)$$

$$F^E_{VVV} = A^3X + B^3(1-X)$$

$$F_V = AX + B(1-X)$$

$$F_B = (1-A)X + (1-B)(1-X)$$

$$F_{VV} = A^2X + B^2(1-X)$$

$$F_{VB} = F_{BV} = A(1-A)X + B(1-B)(1-X)$$

$$F_{BB} = (1-A)^2X + (1-B)^2(1-X)$$

$$F_{VVV} = A^3X + B^3(1-X)$$

$$F_{BVV} = F_{VVB} = A^2(1-A)X + B^2(1-B)(1-X)$$

$$F_{BVB} = A(1-A)^2X + B(1-B)^2(1-X)$$

L_V^E is known as experimental number average sequence lengths of HV units, L_V^R is number average sequence length of randomly distributed HV units in copolymer, k is the ratio between the concentration of HV and HB units, *Pij's* are four conditional probabilities, and r_1r_2 is the reaction index for PHBV.

References

1. Žagar, E.; Kržan, A.; Adamus, G.; Kowalczuk, M. Sequence distribution in microbial poly(3-hydroxybutyrate-co-3-hydroxyvalerate) co-polyesters determined by NMR and MS. *Biomacromolecules* **2006**, *7*, 2210–2216.

2. Kamiya, N.; Yamamoto, Y.; Inoue, Y.; Chujo, R.; Doi, Y. Microstructure of Bacterially Synthesized Poly(3-hydroxybutyrate-co-3-hydroxyvalerate). *Macromolecules* **1989**, *22*, 1676–1682.

3. Wei, L.; Guho, N.M.; Coats, E.R.; McDonald, A.G. Characterization of poly(3-hydroxybutyrate-co-3-hydroxyvalerate) biosynthesized by mixed microbial consortia fed fermented dairy manure. *J. Appl. Polym. Sci.* **2014**, *131*.
